# Melanoma reactive TCR-modified T cells generated without activation retain a less differentiated phenotype and mediate a superior in vivo response

**DOI:** 10.1038/s41598-021-92808-6

**Published:** 2021-06-25

**Authors:** Siao-Yi Wang, Tamson V. Moore, Annika V. Dalheim, Gina M. Scurti, Michael I. Nishimura

**Affiliations:** 1 Department of Medicine, Cardinal Bernardin Cancer Center, Loyola University Chicago, 2160 S 1st Avenue, Maywood, IL 60153 USA; 2 Department of Surgery, Cardinal Bernardin Cancer Center, Loyola University Chicago, Maywood, IL USA

**Keywords:** Cancer immunotherapy, Melanoma

## Abstract

Adoptive T cell therapy with T cell receptor (TCR)-modified T cells has shown promise in treating metastatic melanoma and other malignancies. However, studies are needed to improve the efficacy and durability of responses of TCR-modified T cells. Standard protocols for generating TCR-modified T cells involve activating T cells through CD3 stimulation to allow for the efficient transfer of tumor-reactive receptors with viral vectors. T cell activation results in terminal differentiation and shortening of telomeres, which are likely suboptimal for therapy. In these studies, we demonstrate efficient T cell transduction with the melanoma-reactive TIL1383I TCR through culturing with interleukin 7 (IL-7) in the absence of CD3 activation. The TIL1383I TCR-modified T cells generated following IL-7 culture were enriched with naïve (T_N_) and memory stem cell populations (T_SCM_) while maintaining longer telomere lengths. Furthermore, we demonstrated melanoma-reactivity of TIL1383I TCR-modified cells generated following IL-7 culture using in vitro assays and a superior response in an in vivo melanoma model. These results suggest that utilizing IL-7 to generate TCR-modified T cells in the absence of activation is a feasible strategy to improve adoptive T cell therapies for melanoma and other malignancies.

## Introduction

The introduction of immunotherapies has dramatically improved the treatment of metastatic melanoma over the past two decades. High-dose interleukin-2 (IL-2), immune checkpoint inhibitors, and oncolytic viral therapy have all demonstrated clinical benefit in patients with metastatic melanoma^1–5^. Despite these successes, a significant number of patients fail to respond and the majority of responding patients relapse. Adoptive T cell therapy (ACT) has become an exciting form of treatment for malignant disease. Tumor-infiltrating lymphocytes (TIL) have been shown to induce significant clinical responses in melanoma patients^6–8^. However, the process of TIL preparation is laborious, time-consuming, and dependent on the presence of preexisting anti-tumor reactivity. In addition, not every patient has an accessible tumor to develop TIL cultures. Gene-modified T cells were developed to overcome the limitations of TIL. By transducing cells with tumor-reactive receptors, therapeutic potential is effectively transferred to patient peripheral lymphocytes^9^. We previously described clinical and biological responses in a clinical trial of gene-modified T cells expressing a T cell receptor (TCR) reactive to the melanoma antigen, tyrosinase (TIL1383I)^10^. While this and other trials using melanoma-reactive TCRs show promise, further studies are needed to improve the efficacy and durability of responses of TCR-modified T cells for melanoma.

Studies that characterized T cell subpopulations suggest that stimulation of naïve cells (T_N_) induces differentiation into T memory stem cells (T_SCM_), followed by central memory T cells (T_CM_), and subsequently shorter-lived effector memory T cells (T_EM_) and effector T cells (T_EFF_) in a linear manner^11,12^. Both preclinical and clinical data suggest that less-differentiated populations have superior therapeutic potential. In melanoma and mesothelioma mouse models, T_SCM_ have been shown to be more effective than other subpopulations in mediating tumor regression^13,14^. Clinical trials of chimeric antigen receptor (CAR) T cells for neuroblastoma and B cell malignancies both demonstrate that frequencies of T_SCM_ and T_CM_ correlate with expansion, persistence, and response^15,16^. Most recently, a study demonstrated that a small stem-like population in TIL product was associated with complete response in patients with metastatic melanoma while terminally differentiated TIL were associated poor persistence^17^. Besides T cell phenotype, proliferation potential and the telomere length of cell products have also been correlated with therapeutic efficacy. A trial utilizing TIL in metastatic melanoma patients demonstrated a correlation between clinical response and longer telomere lengths in infused products^18^. Despite these data, current methods of generating both TCR-modified and CAR T cells involve activation through CD3 stimulation to allow for efficient viral gene transfer^19^. This stimulation results in differentiation, shortening of telomeres, and replicative senescence, which are likely suboptimal for therapy^20^.

Interleukin 7 (IL-7) is a T cell homeostatic regulator that induces G0 to G1 progression without inducing cell division or affecting T cell phenotype^21^. Culturing with IL-7 has also been shown to allow for transduction of T cells with lentiviral vectors in the absence of activation^22,23^. In these studies, we show that utilizing IL-7 to generate TIL1383I TCR-modified T cells in the absence of activation results in a cellular product with less-differentiated subpopulations, longer telomere lengths, and antigen-reactivity in vitro and in vivo.

## Results

### Generating TIL1383I TCR-modified T cells in the absence of activation

**Figure 1:**
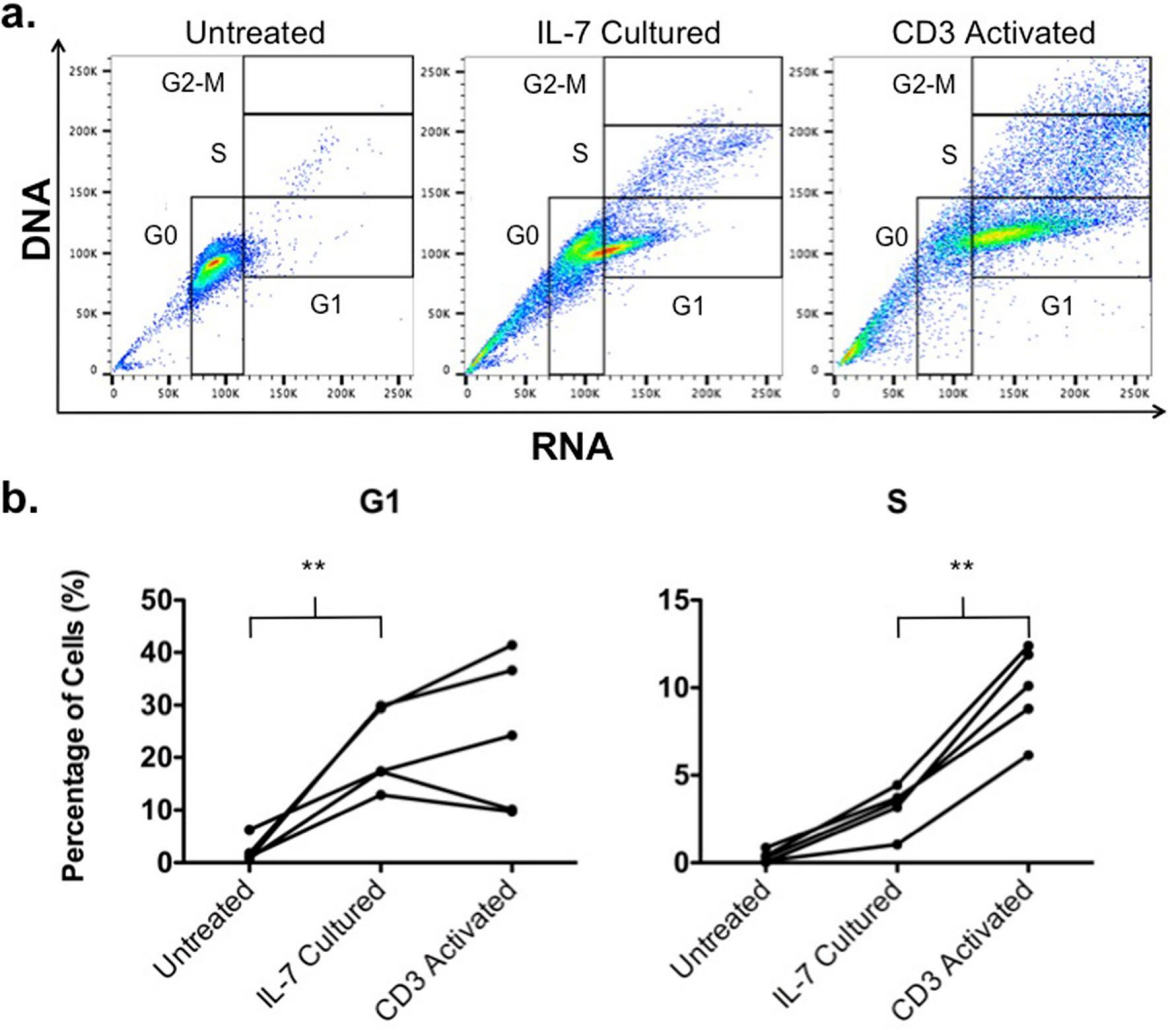
Cell cycle analysis of peripheral blood lymphocytes (PBL)s following no treatment, IL-7 culture, or CD3 activation. (**a**) Flow cytometry was performed on PBLs from an individual donor under each condition after acridine orange staining. This figure is representative of 5 experiments using cells from 5 different donors. (**b**) Percentage of cells in G1 and S phase following each treatment. The graph depicts each data point from 5 donors with lines connecting data points from an individual donor. Statistics were calculated by two-tailed Wilcoxon rank sum test. **P = 0.0079.

As T cell activation likely yields a suboptimal therapeutic product, we aimed to generate melanoma-reactive TCR-modified T cells following culture with IL-7 rather than CD3 stimulation. Prior studies have suggested that progression from G0 to G1 phase of the cell cycle correlates with lentiviral transduction susceptibility^22^. We therefore performed cell cycle analyses using acridine orange on T cells from 5 individual donors following no treatment, IL-7 culture, or CD3 activation. A representative cell staining is depicted in Fig. 1a. Analyses show that IL-7 cultured T cells had significantly more cells in the G1 phase of the cell cycle compared to untreated cells (means of 21% vs. 2%, p = 0.0079; Fig. 1b). CD3 activated T cells had a mean of 28% in G1, which was comparable to IL-7 cultured cells. In contrast, there was no significant difference between IL-7 cultured and untreated T cells in S phase. CD3 activated T cells had significantly more cells in S phase compared to IL-7 cultured cells (means of 10% vs. 3%, p = 0.0079; Fig. 1b). Altogether, these data suggest that culturing with IL-7 induces G0 to G1 progression, potentially rendering T cells susceptible to lentiviral transduction without commitment to S phase and cell division.

**Figure 2:**
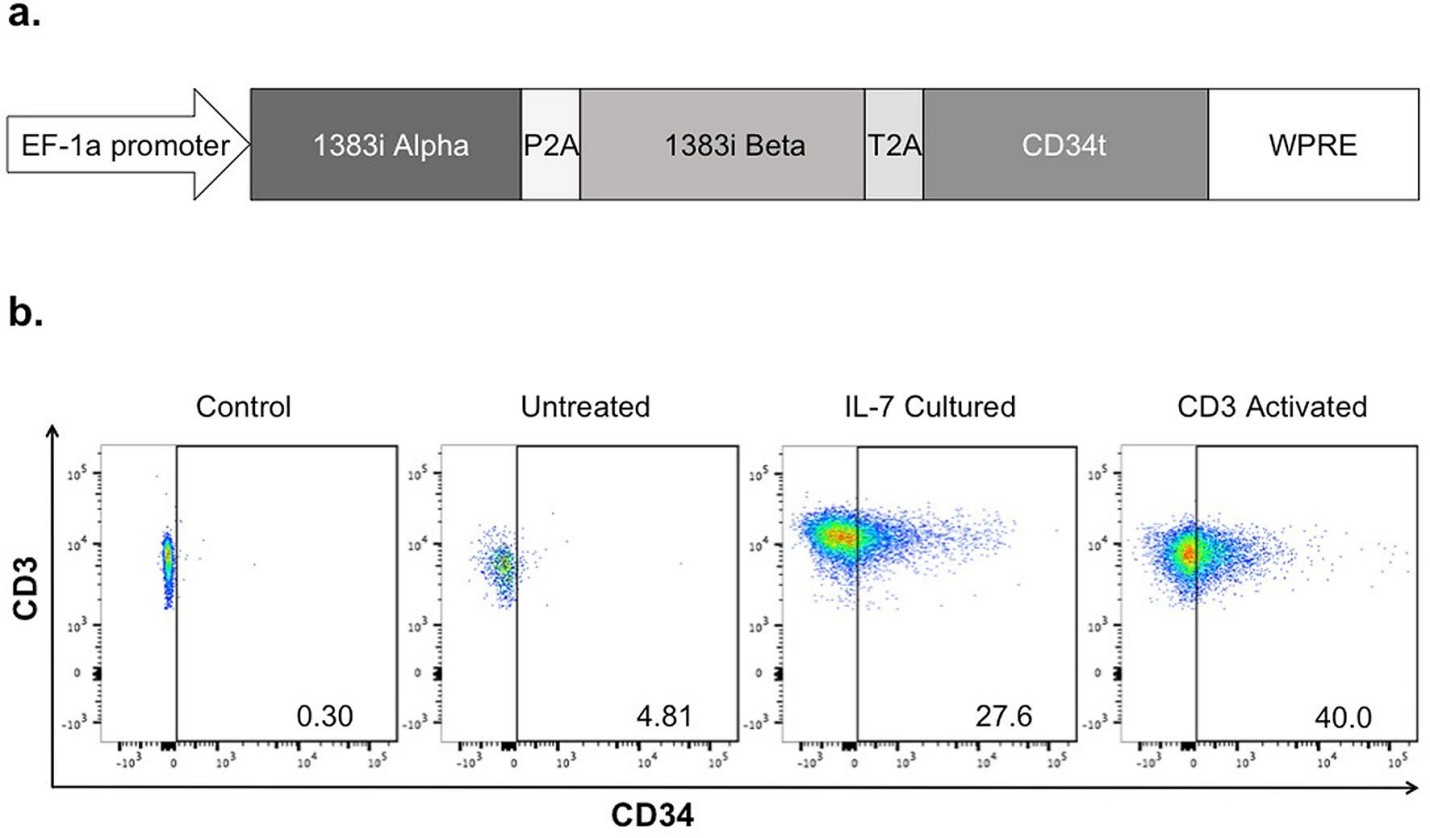
Transduction of PBLs with a lentiviral vector containing a melanoma-reactive T cell receptor (TIL1383I TCR). (**a**) A diagram of the TIL1383I TCR α and β chain genes fused by a P2A self-cleaving peptide linker and attached to a truncated CD34 molecule (CD34t) with a T2A self-cleaving peptide inserted into the pLVX-E1a-N1 lentiviral vector. (**b**) Flow cytometry analysis of CD3^+^ cells transduced with the TIL1383I TCR-containing lentiviral vector either following no treatment, IL-7 culture, or CD3 activation. Untransduced cells without CD34 staining were used as a negative control. These results are representative of 5 experiments using cells from 5 different donors.

We then used our lentiviral vector containing the melanoma-reactive TIL1383I TCR and a non-signaling truncated CD34 transduction marker to transduce T cells, generating TIL1383I TCR-modified T cells (Fig. 2a). As previously described, CD34 expression is used as a marker for gene transfer and additionally allows for the selection of transduced cells using magnetic-based technologies^24^. We transduced T cells from 5 individual donors using our lentiviral vector either after no treatment, IL-7 culture, or CD3 activation. Flow cytometry analysis demonstrated a low level of CD34 expression in untreated CD3^+^ cells. In contrast, CD3^+^ cells demonstrated substantial CD34 expression when transduced after culturing with IL-7 (range of 21.7–55.3%). As expected, activated CD3^+^ cells demonstrated considerable CD34 expression (range of 27.1–62.3%; Fig. 2b). These findings suggest that culturing with IL-7 allows for efficient gene-transfer of TIL1383I TCR to T cells in the absence of CD3 activation.

### Characterizing the phenotypes of TIL1383I TCR-modified T cells generated in the absence of activation

**Figure 3:**
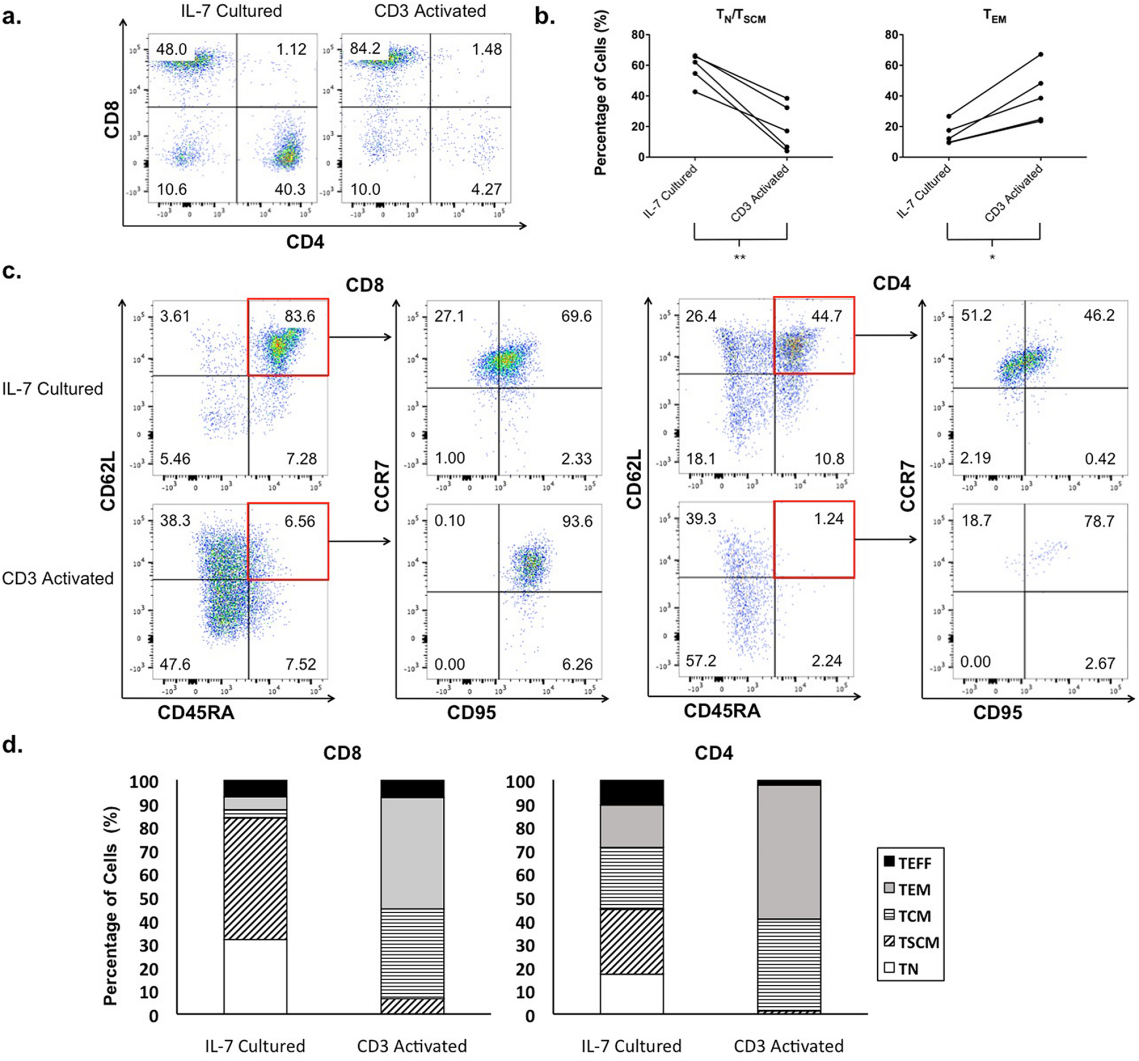
Phenotypic characterization of TIL1383I TCR-modified T cells. (**a**) Flow cytometry analysis depicting CD4^+^ and CD8^+^ populations of CD3^+^CD34^+^ cells transduced either following IL-7 culture or CD3 activation. (**b**) Percentage of T_N_/T_SCM_ (CD62L^+^CD45RA^+^) populations and the T_EM_ (CD62L^-^CD45RA) population in CD3^+^CD34^+^ cells transduced either following IL-7 culture or CD3 activation. The graph depicts each data point from 5 donors with lines connecting data points from an individual donor. Statistics were calculated by two-tailed Wilcoxon rank sum test. *P = 0.0317; **P = 0.0079. (**c**) Flow cytometry analysis depicting the T_N_ (CD62L^+^CD45RA^+^CCR7^+^CD95^-^), T_SCM_ (CD62L^+^CD45RA^+^CCR7^+^CD95^+^), T_CM_ (CD62L^+^CD45RA^-^), T_EM_ (CD62L^-^CD45RA^-^), and T_EFF_ (CD62L^−^CD45RA^+^) populations of CD3^+^CD34^+^CD8^+^ and CD3^+^CD34^+^CD4^+^ cells transduced either following IL-7 culture or CD3 activation. (**d**) Chart depicting the T cell subpopulation composition of CD3^+^CD34^+^CD8^+^ and CD3^+^CD34^+^CD4^+^ cells transduced either following IL-7 culture or CD3 activation. Graphs in (**a**), (**c**), and (**d**) are representative of 5 experiments using cells from 5 different donors.

We next evaluated the phenotypes of TIL1383I TCR-modified T cells generated in the absence of activation to assess for potential therapeutic benefits. As studies have demonstrated that CD4^+^ cells play an important role in the anti-tumor response of gene-modified T cells through both direct cytotoxicity and maintaining persistence, we evaluated the distribution of CD4^+^ and CD8^+^ subsets in CD3^+^ cells transduced either following IL-7 culture or CD3 activation^25^. Although there was heterogeneity between donors, flow cytometry analysis on 5 donors demonstrated that CD3^+^CD34^+^ cells transduced after culturing with IL-7 maintained a relatively balanced CD4^+^/CD8^+^ ratio (CD4^+^ cells ranging from 36.3 to 70.3% and CD8^+^ cells ranging from 30.6 to 48.0%). Interestingly, in the donors we tested, CD3^+^CD34^+^ cells transduced following CD3 activation were predominantly CD8^+^ in our experiments (CD4^+^ cells ranging from 5.6 to 23.3% and CD8^+^ cells ranging from 66.4 to 84.2%; Fig. 3a).

In addition, we assessed the subset composition of TIL1383I TCR-modified T cells generated in the absence of activation as data suggest superior efficacy in less-differentiated populations. The differentiation markers, CD45RA, CD62L, CCR7, and CD95 were evaluated in CD3^+^CD34^+^ cells transduced either following IL-7 culture or CD3 activation. Even with heterogeneity between donors, CD3^+^CD34^+^ cells transduced following IL-7 culture maintained a significantly higher percentage of CD45RA^+^CD62L^+^ cells (T_N_, T_SCM_) when compared to cells transduced following CD3 activation (means of 58.3% vs. 19.7%, p = 0.0079; Fig. 3b). Furthermore, a significant proportion of the CD45RA^+^CD62L^+^ subset of CD3^+^CD34^+^ cells transduced following IL-7 culture was also CCR7^+^CD95^+^ (T_SCM_) (range of 56.9–89.5%). In contrast, CD3^+^CD34^+^ cells transduced following CD3 activation demonstrated a significantly higher percentage of CD45RA^-^CD62L^-^(T_EM_) cells when compared to cells generated following IL-7 culture (means of 40.5% vs. 15.1%, p = 0.0317; Fig. 3b). These findings were consistent when evaluating either CD8^+^ or CD4^+^ subsets individually (Fig. 3c). Notably, there was a lower absolute cell number in the CD4^+^ subset of CD3^+^CD34^+^ cells transduced following CD3 activation. A representation of the phenotypic composition of CD3^+^CD34^+^ cells transduced following IL-7 treatment or CD3 activation is depicted in Fig. 3d. Taken together, these results suggest that generating TIL1383I TCR-modified T cells in the absence of activation leads to a product with a balanced CD4^+^/CD8^+^ ratio and a less-differentiated phenotype, which may be advantageous for therapy.

### Assessing the proliferative potential of TIL1383i TCR-modified T cells generated in the absence of activation

**Figure 4:**
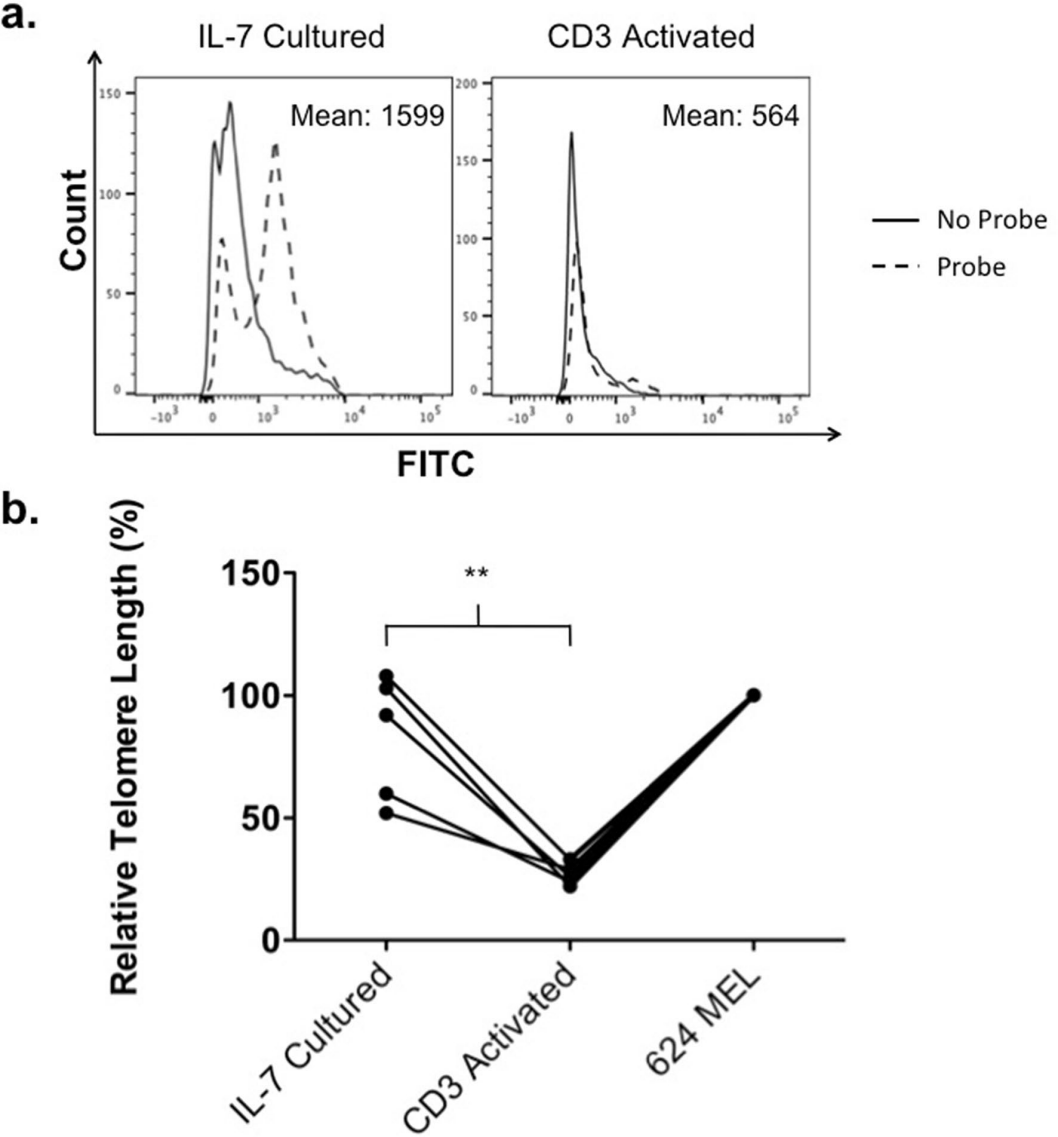
Telomere length analysis of TIL1383I TCR-modified T cells. (**a**) Flow cytometry analysis of depicting fluorescence of cells transduced either following IL-7 culture or CD3 activation after in situ hybridization with a FITC-labelled PNA telomere probe. (**b**) Mean relative telomere length calculated using 624 MEL cells as the control population. The graph depicts each data point from 5 experiments using 5 different donors with lines connecting data points from an individual experiment. Statistics were calculated by two-tailed Wilcoxon rank sum test. ** P = 0.0079.

Studies have shown an established correlation between telomere length and the proliferative potential of peripheral blood lymphocytes (PBLs)^20,26^. We therefore compared the telomere lengths of TIL1383I TCR-modified T cells generated either following IL-7 culture or CD3 activation. We performed fluorescence in situ hybridization (FISH) using a FITC labeled peptide nucleic acid (PNA) probe recognizing the six nucleotide telomere sequence on 5 donors. Although there was variability between donors, we found that CD3^+^CD34^+^ cells transduced following IL-7 culture demonstrated higher fluorescence when compared to cells transduced following CD3 activation, suggesting an increased presence of telomeres (Fig. 4a). Using the melanoma cell line, 624 MEL, as a control, we calculated the relative telomere length of the CD3^+^CD34^+^ cells transduced following each condition. Analysis of the 5 donors demonstrated that the relative telomere length of CD3^+^CD34^+^ cells was significantly higher when transduced following IL-7 treatment compared to CD3 activation (p = 0.0079; Fig. 4b). These findings suggest that TIL1383I TCR-modified T cells generated following IL-7 culture have a higher proliferative potential than cells generated following CD3 activation, which may lead to a more effective therapy.

### Evaluating antigen-specific reactivity of TIL1383I TCR-modified T cells generated in the absence of activation

**Figure 5:**
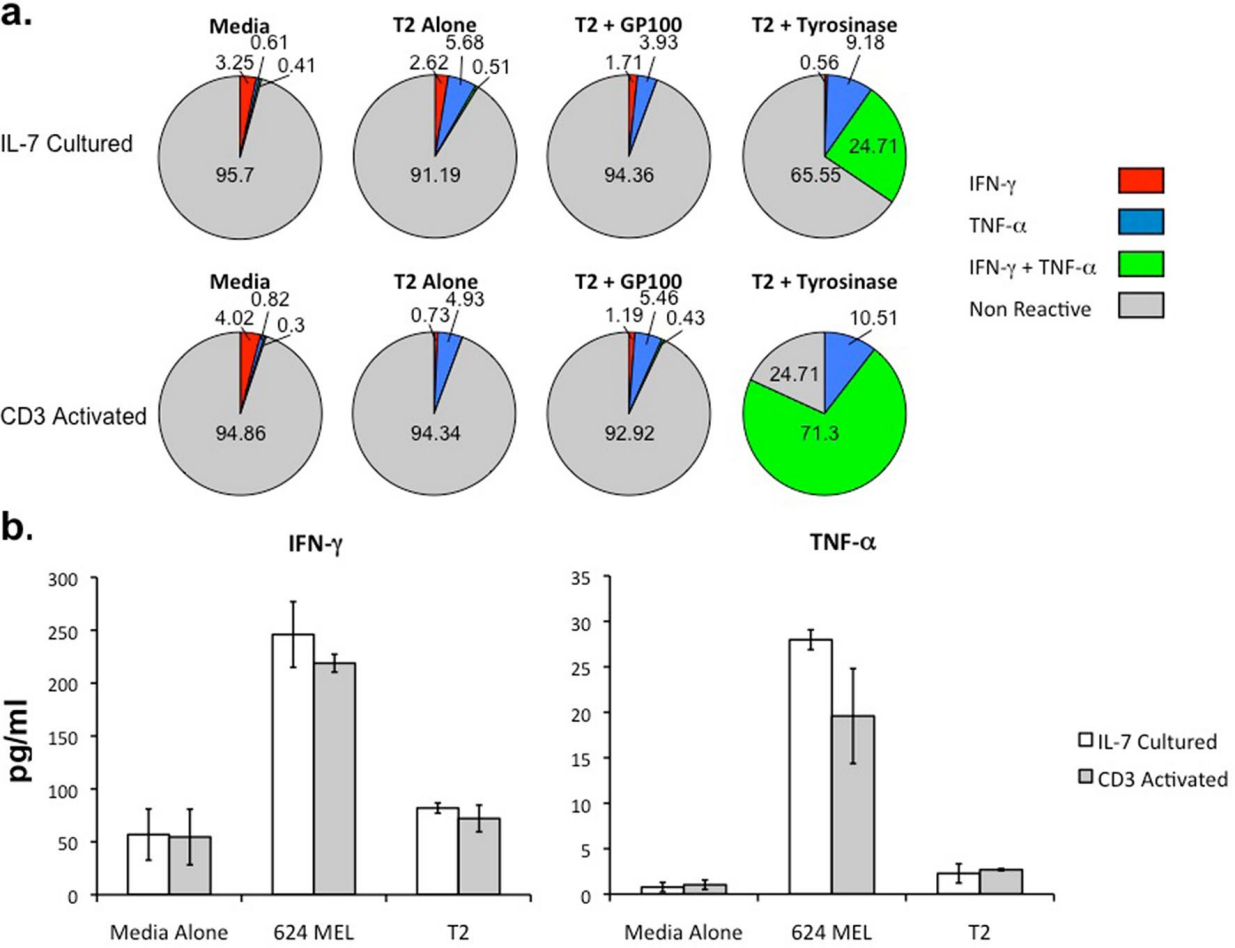
Antigen-specific reactivity of TIL1383I TCR-modified T cells in vitro. (**a**) TIL1383I TCR-modified T cells transduced either following IL-7 treatment or CD3 activation were co-cultured with T2 alone, T2 pulsed with the irrelevant GP100 peptide, or T2 pulsed with tyrosinase peptide in a 1:1 ratio. TIL1383I TCR T cells cultured in media alone were also used as a negative control. Intracellular staining for IFN-α and TNF-β was performed following overnight incubation and analyzed with flow cytometry. Graph depicts percentage of CD3^+^CD34^+^ cells positive for the intracellular cytokines. These results are representative of 3 experiments using 3 different donors. (**b**) TIL1383I TCR-modified T cells transduced either following IL-7 treatment or CD3 activation were co-cultured with 624 MEL cells in a 1:1 ratio. TIL1383I TCR T cells cultured with T2 alone or media alone were used as a negative control. IFN-α and TNF-β release were measured by ELISA. Data represent the mean of triplicate wells with error bars indicating standard deviation. These results are representative of 3 experiments using 3 different donors.

To assess the tyrosinase-specific reactivity of TIL1383I TCR-modified T cells generated in the absence of activation, we performed intracellular cytokine assays following peptide stimulation on 3 donors. CD3^+^CD34^+^ cells transduced following either IL-7 culture or CD3 activation were co-cultured with either unloaded T2 cells, T2 cells loaded with an irrelevant GP100 peptide, or T2 cells loaded with the tyrosinase peptide. Following overnight incubation, intracellular staining of CD3^+^CD34^+^ cells transduced following IL-7 treatment demonstrated a substantial percentage of cells over background producing both IFN–α and TNF-β when stimulated with tyrosinase-loaded T2 cells (Fig. 5a). As expected, CD3^+^CD34^+^ cells transduced following CD3 activation had a high frequency of cells producing both IFN–α and TNF-β when stimulated with tyrosinase-loaded T2 cells. There was expected background cytokine production by cells incubated with media alone, unloaded T2 cells, and T2 cells loaded with an irrelevant GP100 peptide. We then assessed the melanoma reactivity of TIL1383I TCR-modified T cells generated without activation by quantifying cytokine release following stimulation with a melanoma cell line. CD3^+^CD34^+^ cells transduced following either IL-7 culture or CD3 activation from 3 donors were co-cultured with either 624 MEL cells or unloaded T2 cells for 24 h. ELISAs performed on supernatants demonstrated significant presence of IFN-α and TNF-β above background in both cells transduced following IL-7 culture and following CD3 activation (Fig. 5b). These results demonstrate that TIL1383I TCR-modified T cells generated in the absence of activation are reactive against the tyrosinase peptide and a melanoma cell line.

### Evaluating the in vivo anti-melanoma activity of TIL1383I TCR-modified T cells generated in the absence of activation

**Figure 6:**
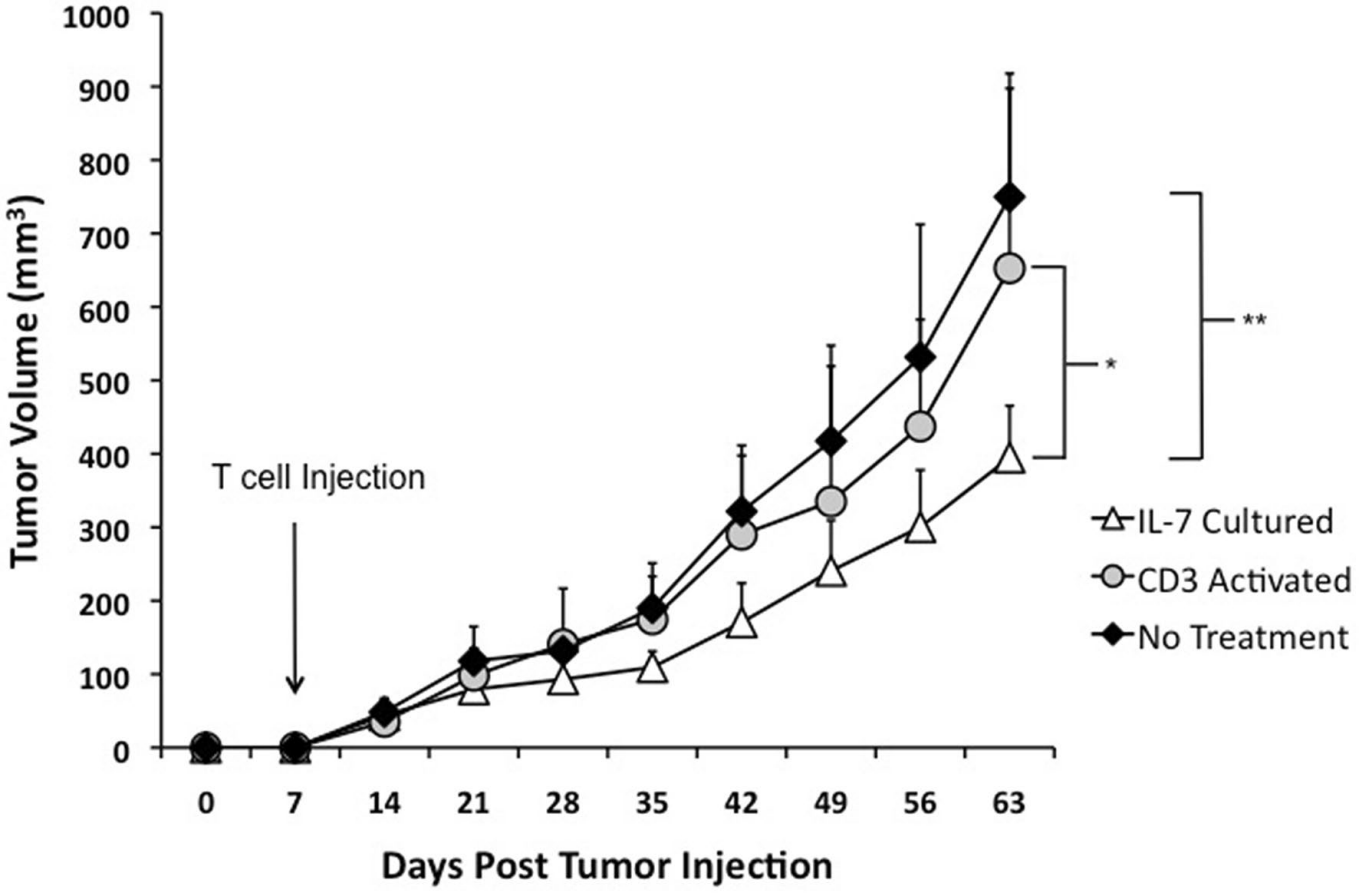
Anti-melanoma activity of TIL1383I TCR-modified T cells in an in vivo model NOD/SCID/γ^−/−^ (NSG) mice were injected subcutaneously with 5 × 10^6^ 624 MEL cells. Mice were treated intravenously upon development of palpable tumor (Day 7) with 5 × 10^6^ TIL1383I TCR-modified cells transduced either following IL-7 culture or CD3 activation. IL-2 (60,000 IU) was then administered through intraperitoneal injection twice a day for 5 days. Tumor-bearing mice receiving no treatment were used as controls. The graph depicts 3 experiments with a total of n = 12 mice per group. Data represent the mean at each time point with error bars indicating standard deviation. Statistical significant differences in tumor size were determined by two-tailed Wilcoxon rank sum test. * P = 0.0480; ** = P < 0.0025.

To assess the in vivo activity of TIL1383I TCR-modified cells, we established tumors through subcutaneous injection of 624 MEL into NOD/SCID/γ^-/-^ (NSG) mice (n = 12 per treatment group). Upon development of palpable tumors (day 7 post injection), 5 × 10^6^ CD3^+^CD34^+^ cells transduced either following IL-7 culture or CD3 activation were adoptively transferred intravenously into tumor-bearing mice. IL-2 was administered twice a day for 5 days following adoptive transfer to enhance T cell responses as previously described and as done in clinical trials^10,27^. Tumor-bearing mice that were untreated or treated with IL-2 alone were used as controls. Mice treated with CD3^+^CD34^+^ cells transduced following IL-7 culture demonstrated a significant delay in tumor progression when compared to untreated mice (p = 0.0025; Fig. 6). Furthermore, mice treated with CD3^+^CD34^+^ cells transduced following IL-7 culture had lower tumor volumes when compared to those treated with activated CD3^+^CD34^+^ cells (p = 0.0480; Fig. 6). In contrast, mice treated with activated CD3^+^CD34^+^ cells did not have a statistically significant difference when compared to untreated mice. IL-2 treatment alone in mice did not have a significant effect on tumor growth (data not shown). Altogether, these data suggest that TIL1383I TCR-modified T cells generated following IL-7 culture have superior anti-melanoma activity in vivo on a per cell basis compared to cells generated following activation.

## Discussion

Adoptive T cell therapy remains a promising form of treatment for metastatic melanoma. As highlighted above, T cell activation used to generate tumor-reactive T cells results in a phenotype that is likely suboptimal for therapy. In addition, TIL, TCR-modified T cells, and CAR T cells all typically undergo in vitro expansion to obtain a sufficient number of cells for clinical response^19^. Continued stimulation during expansion leads to further T cell differentiation, shortening of telomeres, and activation induced cell death (AICD)^18^. As cytokines have been shown to render T cells susceptible to lentiviral transduction in the absence of CD3 stimulation, there have been efforts to generate melanoma-reactive T cells in the absence of activation^22^. One group has previously demonstrated successful transfer of a TCR reactive to the melanoma antigen, MART-1, without prior stimulation of CD3^23^. However, TCR-modified or CAR T cells generated in the absence of activation have yet to be evaluated in vivo*.* This is likely due to the low absolute yield and the inability to select for transduced cells. In these studies, we demonstrate the efficient transfer of TIL1383I TCR and a CD34 marker allowing for magnetic-based sorting without CD3 activation. In addition, we evaluate TCR-modified T cells generated in the absence of activation using an in vivo system for the first time.

IL-2, IL-7, and IL-15 have been demonstrated to enhance lentiviral transduction of T cells without CD3 stimulation^22^. However, IL-2 has been known to result in terminal differentiation and enhance AICD while both IL-2 and IL-15 induce T cell proliferation^28,29^. In contrast, IL-7 is a T cell homeostatic regulator that promotes T cell survival without stimulating cell division^21^. Cell cycle analysis in our studies confirmed that IL-7 culture transitions lymphocytes from G0 to G1 without commitment to S phase and therefore proliferation (Fig. 1). Thus, we utilized IL-7 in our studies to facilitate gene transfer in attempt to preserve maximal immune-competence.

Using our lentiviral construct, we were able to efficiently generate TIL1383I TCR-modified T cells following IL-7 culture (Fig. 2b). In the donors we tested, cells transduced following IL-7 culture maintained a relatively balanced CD4^+^/CD8^+^ ratio compared to the low CD4^+^/CD8^+^ ratio we observed in activated cells. Prior studies from another group found a similarly high proportion of CD8^+^ cells when transducing stimulated T cells^30^. Infusion products used in different CD19 CAR T cell trials also frequently skewed toward an either predominantly CD4^+^ or CD8^+^ population^31–33^. Although the causes are not completely clear, differences in transduction and in vitro expansion protocols, including methods of T cell stimulation, cytokines, feeder cells, viral vectors, and length of expansion, all may account for inconsistent CD4^+^/CD8^+^ ratios. Patient sex, age, ethnicities, or prior exposures may also play a role. As variability in the infusion product likely accounts for the unpredictable clinical responses to adoptive cell therapy, there have been efforts in generating a more consistent product. Studies have shown that an infusion product formulated with defined CD4^+^ and CD8^+^ subsets in a 1:1 ratio enhances in vivo anti-tumor activity^25,34^. This has led to a CAR T trial in which CD4^+^ and specific CD8^+^ subsets were purified and transduced separately before formulated in a 1:1 ratio^35–37^. Although the trial demonstrated significant clinical responses, the manufacturing process remains laborious. Generating TCR-modified T cells following IL-7 culture appears to be a straightforward strategy to achieve a similar CD4^+^/CD8^+^ composition without the potential consequences of T cell activation.

As evidence suggests that less-differentiated T cells have higher therapeutic potential, there are ongoing efforts to preserve less-differentiated phenotypes of TCR-modified or CAR T cells. The group that formulated an infusion product with a defined CD4^+^/CD8^+^ ratio used a purified CD8^+^ T_CM_ starting population for transduction^35–37^. In addition, the use of IL-7 and IL-15 during in vitro expansion has been used to maintain less-differentiated T cell populations^16,38^. However, both of these strategies continue to rely on CD3 stimulation for efficient gene transfer. Even when cell products were generated from a less-differentiated CD8^+^ T_CM_ population, the patients received a product that consisted of CD8^+^ cells that were mostly T_EM_^35,37^. This is consistent with our studies that demonstrate significant T cell differentiation following activation (Fig. 3b–d). In addition, a recent study evaluating TCR-modified T cells specific to the cancer-testis antigen, NY-ESO-1, following expansion with IL-7 and IL-15 demonstrated a limited enrichment of less-differentiated cells and no absolute benefit^39^. By transducing T cells in the complete absence of activation, we obtain TIL1383I TCR-modified T cells with preserved T_N_ and T_SCM_ populations and retained telomere lengths (Figs. 3 and 4).

We confirmed the melanoma-specific reactivity of TIL1383I TCR-modified T cells generated following IL-7 culture using both in vitro and in vivo systems. Intracellular cytokine assays demonstrated cytokine production when TIL1383I TCR-modified cells generated in the absence of activation were incubated with tyrosinase-loaded targets. However, cytokine production of activated TIL1383I TCR-modified T cells appeared to be more robust in this assay (Fig. 5a). This is likely because the cells generated following IL-7 culture are enriched with T_N_ and T_SCM_ subpopulations, which require more time to react in vitro, but have been shown to have a higher proliferative potential and be a superior therapy in vivo^13^. The results following overnight incubation are not reflective of the overall therapeutic potential of TIL1383I TCR-modified T cells generated without activation. Indeed, these cells demonstrated cytokine release comparable to activated TIL1383I TCR-modified T cells when ELISAs were performed on supernatants after culturing with a melanoma cell line for 24 h (Fig. 5b). In addition, we found a superior anti-melanoma response when mice were treated with TIL1383I TCR-modified T cells generated following IL-7 culture compared to mice treated with activated cells (Fig. 6).

Even with efficient gene-transfer of TIL1383I TCR without CD3 stimulation, the percentage of transduced cells was lower than a typical infusion product. However, our CD34t transduction marker permits clinical-grade magnetic selection, resulting in a highly pure transduced product with enhanced tumor-reactivity^24^. In vitro expansion is commonly used to increase the percentage of transduced cells and obtain the required number of cells for infusion, but this comes at the expense of telomere shortening and reduced proliferative potential^18^. It would be difficult to obtain comparable absolute numbers of gene-modified cells typical for infusion without in vitro expansion. However, TIL1383I TCR-modified T cells generated in the absence of activation have desirable qualities for therapy, so the number of cells required to see a clinical response may not be as high. Our in vivo experiments used a number of cells that was 50% of what we previously used when evaluating TCR-modified T cells in vivo to assess for tumor reactivity at a lower than standard dose^40^. Indeed, our in vivo experiments demonstrate significant melanoma reactivity at a lower than standard cell number, justifying a lower starting dose for future clinical trials. Furthermore, generating a potent therapy without in vitro expansion would shorten production time and permit more timely treatment.

In conclusion, we demonstrate efficient generation of melanoma-reactive TIL1383I TCR-modified T cells in the absence of activation by utilizing IL-7 treatment and a lentiviral vector. TIL1383I TCR-modified T cells generated following IL-7 culture have a balanced CD4^+^/CD8^+^ ratio, less-differentiated phenotype, and increased proliferative potential. These TIL1383I TCR-modified T cells generated in the absence of activation have anti-melanoma reactivity both in vitro and in vivo. Studies investigating the in vivo persistence of TIL1383I TCR-modified T cells generated without activation and the phenotype of the persisting cells are currently underway. A growing body of data suggests that less-differentiated phenotypes and longer telomeres correlate with clinical response. Thus, generating TIL1383I TCR-modified T cells in the complete absence of activation is a feasible strategy to improve therapeutic potential. These findings may be applied to other tumor reactive TCRs and CARs and improve adoptive T cell therapy for melanoma and other malignancies.

## Materials and methods

### Cells lines

T2, a human TAP-deficient HLA-A2^+^ cell line, and HEK 293 T were obtained from ATCC (Rockford, MD). 624 MEL, a melanoma cell line was previously described (Bethesda, MD)^41^. GPRTG, a 293 T-based packaging cell line for production of lentiviral vector particles, was obtained from the National Gene Vector Biorepository (Indiana University, Indianapolis, IN)^42^. T2 cells were maintained in RPMI (Thermo Fisher, Walther, MA) with 10% heat-inactivated fetal bovine serum (HI-FBS; Tissue Culture Biologics, Long Beach, CA). All other cell lines were maintained in DMEM (Thermo Fisher) with 10% HI-FBS. 1 ng/mL doxycycline (Clontech Laboratories, Palo Alto, CA) was added to DMEM for GPRTG cells. Cells were maintained at 37 °C in a humidified 5% CO_2_ incubator.

### T cells

Peripheral blood lymphocytes (PBLs) from independent normal human donors were isolated from purchased apheresis products (Key Biologics, Memphis, TN) using Ficoll-Hypaque (Sigma-Aldrich, St. Louis, MO) density gradients^40,43^. T cells were activated by stimulating PBLs from an individual donor with 50 ng/mL anti-CD3 mAb (Miltenyi Biotec, Bergisch Gladbach, Germany), 300 IU/mL recombinant human IL-2 (rhIL-2; Novartis Pharmaceuticals, East Hanover, NJ), and 100 ng/mL recombinant human IL-15 (rhIL-15; Biological Resources Branch, National Cancer Institute, Bethesda, MD) for three days prior to transduction^43^. Alternatively, PBLs from the same donor were cultured with 20 ng/mL recombinant human IL-7 (rhIL-7; Biological Resources Branch, National Cancer Institute) for seven days prior to transduction.

### Cell cycle analysis

Cell cycle analysis was performed using acridine orange as previously described^44^. 10^6^ cells were resuspended in 0.1 ml of PBS. 0.5 ml of Buffer I (20 mM Citrate–Phosphate, pH 3.0, 0.1 mM EDTA, 0.2 M Sucrose, 0.1% Triton X-100) was added to the suspension and gently agitated. 2 mg/ml acridine orange (Sigma-Alrich) was diluted 1:100 in Buffer II (10 mM Citrate–Phosphate, pH 3.8, 0.1 M NaCl). 0.5 ml of Buffer II containing acridine orange was subsequently added to the suspension and gently agitated. Cell luminescence was measured using the LSR Canto flow cytometer (BD Biosciences, San Jose, CA) and data was analyzed with FlowJo software V10.1 (BD Biosciences, https://www.flowjo.com). The fraction of cells in G0, G1, S, and G2/M phases of the cell cycle was determined by DNA and RNA contents.

### Lentivirus production

A lentiviral vector was constructed to include the melanoma antigen (tyrosinase)-reactive TIL1383I TCR, and a truncated CD34 molecule (CD34t) as a transgene expression marker as previously described^24^. The pLVX-E1a-N1 (Clontech, Mountain View, CA) vector was modified to include the TCR alpha chain, P2A self-cleaving linker, TCR beta chain, T2A linker, and CD34t. This modified vector was used to generate a stable GPRTG lentiviral producer cell line as follows. Viral supernatants were produced by transfecting 293 T cells with the pLVX-TIL1383I TCR plasmid using Lenti-X single shots following the manufacturer’s directions (Takara, Mountain View CA). GPRTG cells were spinoculated (1000 X G for 2 h at 32 °C) with viral supernatants containing 8 µg/ml of polybrene (Sigma-Alrich), cultured for an additional 72 h, and resuspended in DMEM with doxycycline. TIL1383I TCR lentiviral supernatants for T cell transductions were produced as follows. Transduced GPRTG cells were seeded in doxycycline-free DMEM in 10 cm tissue culture dishes. DMEM was replaced with Freestyle 293 expression media (Gibco, Gaithersburg, MD) 24 h later. TIL1383I TCR lentiviral supernatant was then collected following a 48 h incubation.

### Lentiviral transduction of T cells

PBLs from an individual donor that were either cultured with IL-7 or activated with anti-CD3, IL-2, and IL-15 were resuspended in TIL1383I TCR lentiviral supernatant containing 8 µg/ml of polybrene and plated in 24 well plates. Plates were spinoculated at 2000 X G at 32 °C for 2 h. TIL1383I TCR-transduced cells were harvested and resuspended in RPMI supplemented with either IL-7 or IL-2 and IL-15.

### T cell phenotypic analysis

Evaluation of T cell surface markers was performed via immunofluorescence staining and analyzed via flow cytometry. Monoclonal antibodies (mAbs) used to characterize transduced T cells include: anti-CD3-APC Cy7, anti-CD4-APC, anti-CD8-PerCP/Cy5.5, anti-CD34-PE, anti-CCR7-BV785, anti-CD45RA-PE Cy7 (Biolegend, San Diego, CA), and anti-CD62L-BV650, anti-CD95-FITC (BD Biosciences). Flow cytometry was performed using the LSR Fortessa flow cytometer (BD Biosciences) and data was analyzed with FlowJo software V10.1.

### Telomere analysis

The flow-FISH method using the telomere PNA Kit/FITC for Flow Cytometry Kit (Dako, Glostrup, Denmark) was used to analyze telomere length per manufacturer’s protocol. 624 MEL cells were used as the control. Fluorescence was measured using an LSR Canto flow cytometer. Data was analyzed with FlowJo software V10.1.

### Cytokine production and release assays

Antigen reactivity of transduced cells was measured through cytokine production and cytokine release as previously described^40^. Tyrosinase-specific reactivity was assessed through an intracellular cytokine assay as follows. Gp100:209–217 (gp100) and tyrosinase:368–376 (tyrosinase) peptides were obtained from Synthetic Biomolecules (San Diego, CA). T2 cells were pulsed with 10 µg/ml of peptide for 2 h prior to co-culture. 10^5^ TIL1383I TCR-modified cells were co-cultured with the targets at a 1:1 ratio in a 96 well U-bottom tissue culture plate in 200 µl media. Following overnight incubation, Brefeldin-A and Monensin (Biolegend) were added to the culture. After five hours, cells were stained for surface markers for 20 min at room temperature. Cells were fixed, permeabilized, and stained for intracellular IFN-α and TNF-β. Data was collected using an LSR Fortessa flow cytometer and analyzed with FlowJo software V10.1. Alternatively, melanoma reactivity was assessed through a cytokine release assay as follows. 10^5^ TIL1383I TCR-modified cells were co-cultured in triplicate with 624 MEL cells at a 1:1 ratio in a 96 well U-bottom tissue culture plate in 200 µl media. Supernatants were harvested following incubation for 24 h. Cytokine release was quantified by sandwich ELISA using monoclonal antibodies to IFN-α or TNF-β (Biolegend).

### Melanoma xenograft model

8–12-week old NOD/SCID/γ^−/−^ (NSG) male and female mice (Jackson Laboratory, Bar Harbor, ME) were treated under an approved IACUC protocol and complied with ARRIVE guidelines. Mice were injected subcutaneously with 5 × 10^6^ 624 MEL cells into flanks, forming palpable tumors by day 7. At 7 days post-challenge, 5 × 10^6^ TIL1383I TCR-modified cells were injected intravenously in 100 µl of PBS. Intraperitoneal injections of IL-2 (60,000 IU) were administered twice a day for 5 days as previously described^27^. Tumor bearing mice either treated with IL-2 alone or untreated were used as controls. Tumor size in 3 perpendicular dimensions was measured twice a week using calipers. Two-tailed Wilcoxon rank sum test was used to determine significant difference between treatment groups.

### Ethics approval

All applicable international, national, and/or institutional guidelines for the care and use of animals were followed. All animal studies were conducted under approved Loyola University Institutional Animal Care and Use Committee (IACUC) protocols and complied with ARRIVE guidelines. All recombinant DNA and lentiviral transductions work was done under approved Loyola Institutional Biosafety Committee (IBC) protocols. This article does not contain any studies with human participants performed by any of the authors. All human materials used were from de-identified apheresis products purchased from commercial sources.

## Data Availability

The data that support the findings of this study and materials are available from the corresponding author, SW, upon reasonable request.
